# lncRNA MALAT1 Promotes Renal Fibrosis in Diabetic Nephropathy by Targeting the miR-2355-3p/IL6ST Axis

**DOI:** 10.3389/fphar.2021.647650

**Published:** 2021-04-29

**Authors:** Haozi Huang, Guowei Zhang, Zhenying Ge

**Affiliations:** ^1^ Department of Endocrinology, Huaihe Hospital of Henan University, Kaifeng, China; ^2^ Intensive Care Unit, Adult Cardiovascular Surgery, Fuwai Central China Cardiovascular Hospital, Zhengzhou, China; ^3^ Basic Medical College, Henan University, Kaifeng, China

**Keywords:** diabetic nephropathy, lncRNA MALAT1, miR-2355-3p, IL6ST, STAT3 pathway

## Abstract

Long noncoding RNA (lncRNAs) metastasis–associated lung adenocarcinoma transcript 1 (MALAT1) has been reported in diabetic nephropathy (DN) about its effect on podocyte function and cell heat shock induced by hyperglycemia. However, the biological mechanism of MALAT1 regulating DN fibrosis needs further study. In this study, SD rats were administrated with streptozotocin (STZ) to establish a diabetes model. *In vitro*, human renal tubular epithelial cells (HK-2 and 293T) were treated with high glucose (HG). Here, we found that MALAT1 was upregulated in renal tissues of diabetic rats and HG-treated cells, and HG treatment promoted cell proliferation and invasion. MALAT1 overexpression aggravated protein levels of collagen I (col I), collagen IV (col IV), fibronectin (FN), and laminin (LN) in HK-2 cells, while MALAT1 knockdown exerted the opposite effect. Moreover, the luciferase reporter gene and pull-down assays demonstrated that MALAT1 interacted with miR-2355-3p. The miR-2355-3p level was downregulated in diabetic rats and HG-treated cells, and MALAT1 overexpression inhibited the miR-2355-3p level. Bioinformatics prediction and luciferase reporter gene assay revealed that interleukin 6 signal transducer (IL6ST) was a target of miR-2355-3p. In addition, miR-2355-3p overexpression attenuated fibrosis-related gene levels in HG-treated cells by inhibiting IL6ST expression and inactivating the recombinant signal transducer and activator of the transcription 3 (STAT3) signaling pathway. Knockdown of miR-2355-3p reversed the inhibitory effect of MALAT1 knockdown on IL6ST, col I, col IV, FN, and LN protein levels in HG-induced cells. Overexpression of MALAT1 aggravated cell damage in HG-induced cells *via* the miR-2355-3p/IL6ST/STAT3 signaling pathway. Finally, enhanced renal fibrosis and kidney tissue damage were observed in diabetic rats. In conclusion, MALAT1 overexpression may enhance renal fibrosis in diabetic rats and cell damage in HG-induced HK-2 cells *via* the miR-2355-3p/IL6ST axis, which provides a new perspective of DN treatment.

## Introduction

Diabetic nephropathy (DN), one of the most important complications of diabetic patients, is the main cause of death. Long-term hyperglycemia is associated with micro- and macrovascular complications that affect multiple organs, including the eyes and kidneys (Fox et al., 2015; Gao et al., 2018a; Muthuppalaniappan et al., 2019; Ni et al., 2019). DN has become one of the most common complications in diabetic patients (Doi et al., 2008; Kanasaki et al., 2013; Thomas and Karalliedde, 2019). It is reported that about 1/4 of diabetic patients eventually develop DN (Thomas and Karalliedde, 2019).

Dipeptidyl peptidase-4 (DPP-4) inhibitors and sodium glucose cotransporter-2 (SGLT-2) inhibitors, oral drugs for the treatment of patients with type 2 diabetes mellitus (T2D), are mainly used to control blood glucose (Shimizu et al., 2019). Both of them play a protective role in the progression of renal disease and play an anti-fibrosis role in DN (Gupta and Sen, 2019). Angiotensin-converting enzyme (ACE) inhibitors (ACEIs) and AT1 receptor antagonists (ARBs) are the first-line drugs to reduce the progression of end-stage renal disease in patients with diabetes. Angiotensin-converting enzyme inhibitors and N-acetyl-seryl-aspartyl-lysyl-proline (AcSDKP) inhibit renal metabolism–related mesenchymal transformation and reduce the accumulation of collagen-I and fibronectin (Srivastava et al., 2020a). Although blocking the renin–angiotensin system can be used to control blood pressure and statins to lower blood lipids, the number of DN patients requiring hemodialysis has continued to increase over the past decade (Kanasaki et al., 2013). Therefore, it is urgent to find new targets for DN treatment.

Long noncoding RNAs (lncRNAs) are a large class of 200-nt long noncoding transcripts that lack the ability of protein coding. lncRNA metastasis–associated lung adenocarcinoma transcript 1 (MALAT1) was first found in early-stage non–small-cell lung cancer, and it could be a diagnostic factor to observe the metastasis of early non–small-cell lung cancer (Ji et al., 2003). MALAT1 encodes an 8.7-kb exon transcript and is highly conserved in mammals. In addition, MALAT1 is highly expressed in the brain, kidney, and other tissues (Zhang et al., 2017; Gordon et al., 2018; Guo et al., 2019a). It was reported that lncRNA MALAT1 enhances apoptosis and oxidative stress of lens epithelial cells in diabetic cataract through activation of the p38 mitogen-activated protein kinase (p38MAPK) signaling pathway (Gong W. et al., 2018). Moreover, human gastric smooth muscle cells were transfected with siRNAs against MALAT1 to inhibit MALAT1 levels, and knockdown of MALAT1 inhibited cell migration and induced cell apoptosis (Gong Y. et al., 2018). A large number of studies showed that lncRNAs play an important role in the occurrence and development of DN (Gong Y. et al., 2018; Li et al., 2018). The inhibitory effect of lncRNAs on DN is mainly through the inhibition of renal cell proliferation and fibrosis. It was reported that lncRNA NR_038323 and lncRNA growth arrest–specific transcript 5 (GAS5) suppress renal fibrosis in DN (Ge et al., 2019a; Ge et al., 2019b). In contrast, it was reported that lncRNA–linc00968 accelerated the proliferation and fibrosis of DN cells (Li et al., 2018). MALAT1 was also reported in DN, and early intervention of MALAT1 knockdown partially restored podocyte function and prevented the accumulation of β-catenin nucleus (Hu et al., 2017). It has been previously reported that lncRNA MALAT1 regulates renal tubular epithelial pyroptosis in DN by regulating miR-23c targeting ELAVL1 (Li et al., 2017a). Inspired by these findings, we explored other mechanisms by which lncRNA MALAT1 regulates the pathogenesis of DN to provide a novel therapeutic target for DN.

MicroRNAs (miRNAs) are a class of endogenous, noncoding small RNAs (22 nucleotides) that pair with 3′untranslated regions (3′UTRs) and regulate gene expression. Emerging evidence has shown that miRNAs play important roles in cell proliferation, invasion, and apoptosis in DN progression (Wang et al., 2016; Sun et al., 2018). In DN, downregulation of microRNA-21 reduces inflammation by reducing the expression of tissue inhibitors of metalloproteinase 3 (TIMP3) (Chen et al., 2018). Knockdown of miR-133b and miR-199b attenuates the epithelial-mesenchymal transformation and renal fibrosis induced by TGF-271 by targeting SIRT1 in diabetic nephrosis (Sun et al., 2018). miR-451 inhibited the expression of pro-inflammatory molecules mediated by NF-kB *via* inhibiting large multifunctional protease 7 (LMP7) (Sun et al., 2016). It was reported that miR-2355-3p regulated intervertebral disc degeneration by targeting ERFFI1 (the product of mitogen-inducible gene 6) (Guo et al., 2019). However, miR-2355-3p is rarely reported in DN progression. StarBase (http://starbase.sysu.edu.cn/) was used to predict that miR-2355-3p could bind with MALAT1 on chr11:65272680–65272699 [+]. Subsequently, we explored the role of miR-2355-3p in DN progression.

Inspired by these findings, we conjectured that MALAT1 may regulate DN progression by adsorbing miR-2355-3p. To test our hypothesis, we examined the expression of lncRNA MALAT1 in high glucose (HG)–treated HK-2 and its effects on various fibrin levels and cell behaviors. In addition, we constructed the STZ rat model *in vivo* to verify the mechanism of MALAT1 regulating DN progression.

## Materials and Methods

### Animal Studies

Twenty Sprague Dawley (SD) (6–8 weeks old) rats were provided by the Animal Center of Huaihe Hospital of Henan University, China. All experiments were approved and performed according to the guidelines of the Ethics Committee of Huaihe Hospital of Henan University. Streptozotocin (STZ) (Sigma, S0130, St. Louis, MO, United States) dissolved in citrate buffer was used to induce diabetes of rats. STZ was injected intraperitoneally on an empty stomach, with a continuous injection of 50 mg/kg for 5 days, and sodium citrate (SC) was used as control. Two consecutive fasting blood glucose levels above 200 mg/dl were considered diabetes. Rats were kept in a light/dark cycle for 12 h and could eat and drink freely. To observe the effect of miR-2355-3p and MALAT1 on renal fibrosis, the rats were intraperitoneally injected with MALAT1 siRNA, LNA-anti-miR-2355-3p, and scramble (a negative control of MALAT1 siRNA or LNA-anti-miR-2355-3p) every day. LNA-anti-miR-2355-3p (40 μg) (IDT Inc., Coralville, IA, United States) was given twice weekly (2-week and 12-week group). Rats were injected with MALAT1 siRNA (IDT Inc., Coralville, IA, United States) *via* the tail vein at 25 μg each 2 weeks for 4 weeks. After 12 weeks, the rats were euthanized, and the kidney (including renal cortex), heart, liver, bladder, and peritoneum tissues were taken for follow-up analysis.

### Cell Lines and Cell Culture

The human renal epithelial cell lines (HK-2 and 293T) utilized in the present study were purchased from the Institute of Cell Research, Chinese Academy of Sciences (Shanghai, China). The authenticity of cell lines has been verified by short tandem repeat analysis. All cells were cultured in Roswell Park Memorial Institute 1640 medium (RPMI-1640, Gibco, BRL, Carlsbad, CA, United States) supplemented with 10% fetal bovine serum (Sigma-Aldrich, St. Louis, MO, United States), then incubated in a 37°C incubator with a humidified atmosphere of 5% CO_2_. Subsequently, HK-2 and 293T cells were treated with 5 mM D-glucose (NG), 30 mM D-glucose (HG), or mannitol (a negative control of HG) to explore the effect of HG on cell behaviors.

### Analysis of Renal Function and Physiological Parameters

The blood glucose levels of rats were measured by a glucose assay kit (Thermo Fisher Scientific, Waltham, MA, United States) according to the manufacturer’s instructions. Bromocresol green (BCG) albumin assay kit (Sigma, St. Louis, MO, United States) was used to measure urine albumin levels. The high-performance liquid chromatography (HPLC) method was used to determine serum and urine creatinine levels (Thienpont et al., 1995). The ratio of urinary albumin to creatinine (ACR) was calculated by using the method described previously (Zhan et al., 2015).

### Reverse Transcription-qPCR (RT-qPCR)

Total RNA was extracted from cells and tissues according to the instruction of the TRIzol reagent (Invitrogen, Carlsbad, CA, United States) and purified with the RNeasy Maxi kit (Qiagen, Dusseldorf, Germany) according to the manufacturer’s protocol. Total RNA (1 µg) was then used for cDNA synthesis with a Transcriptor First Strand cDNA Synthesis Kit (Roche, IN, United States). RT-qPCR was performed with the SYBR^®^ Green PCR Master Mix kit (Qiagen, Dusseldorf, Germany) on an ABI 7500 real-time PCR system (Applied Biosystems, Waltham, MA, United States), and the detailed process was as follows: 95°C for 10 min followed by 35 cycles of 95°C for 20 s, then 56°C for 10 s, and 72°C for 15 s. Finally, the relative expression levels of lncRNA MALAT1, miR-2355-3p, and IL6ST were calculated by using the 2^−ΔΔCt^ method. We used 18 s (for lncRNA), U6 (for miRNA), or GAPDH as endogenous controls, respectively. The sequences of primers were as follows: MALAT1 forward: 5′-CTT AAG CGC AGC GCC ATT TT-3′ and reverse: 5′-CCT CCA AAC CCC AAG ACC AA-3ʹ; miR-2355-3p forward: 5′-CTG AGG GAT CCC CAG ATA CAA TGG-3′ and reverse: 5′-GTG CAG GGT CCG AGG T-3′; IL6ST forward: 5′-GTG TGA AAG CAG CAA AGA GGC-3′ and reverse: 5′-CTG GAG GTA CTC TAG GTA TAC-3’′; GAPDH forward: 5′-ATC CCA TCA CCA TCT TCC AG-3′ and reverse: 5′-CAC ACC CAT GAC GAA CAT GGG-3′; 18 s forward: 5′-CGA AAG CAT TTG CCA AGA AT-3′ and reverse: 5′-AGT CGG CAT CGT TTA TGG TC-3ʹ; U6 forward: 5′-GCT TCG GCA CAT A-3′ and reverse 5′-ATG GAA CGC TTC ACG A-3′.

### Cell Transfection

miR-2355-3p mimic (100 nM), NC mimic (a negative control for miR-2355-3p mimic), miR-2355-3p inhibitor (100 nM), NC inhibitor (a negative control for miR-2355-3p inhibitor), IL6ST siRNA (100 nM), MALAT1 siRNA (100 nM), and NC siRNA or scramble (a negative control for IL6ST siRNA or MALAT1 siRNA) were synthesized by Invitrogen (Carlsbad, CA, United States). MALAT1 overexpression plasmid (pcDNA-lncRNA MALAT1) was synthesized by Thermo Fisher Scientific (Waltham, MA, United States), and pcDNA3.1 (Vector, a negative control for pcDNA-lncRNA MALAT1) was purchased from Invitrogen (Carlsbad, CA, United States). The transfection and co-transfection of these oligonucleotides and overexpression vectors were carried out with Lipofectamine Plus reagent (Invitrogen, Carlsbad, CA, United States) according to the manufacturer’s instructions.

### Western Blotting

RIPA lysis buffer (CW Biotech, Beijing, China) was used to lyse cells and extract total protein. The protein concentration was measured by the BCA protein assay kit (Thermo Fisher Scientific, Waltham, MA, United States). 10% SDS-PAGE was used to separate protein samples in equal amounts. Next, we transferred the complex onto a nitrocellulose membrane (General Electric Co., United States). After blocked with 5% skim milk, the blots were probed with primary antibodies. Following washed with TBST, the horseradish peroxidase–conjugated goat anti-mouse IgG H&L (HRP) (ab205719, 1:1000, Abcam, Cambridge, United Kingdom) were added. Next, we incubated the membrane for 1 h at room temperature. The primary antibodies used in this study included mouse anti-collagen I (col I) antibody (ab6308, 1:300, Abcam), mouse anti-collagen IV (col IV) antibody (ab86042, 1:400, Abcam), mouse anti-fibronectin (FN) antibody (ab154210, 1:300, Abcam), mouse anti-laminin (LN) antibody (FK-CV0821P, Shenzhen Fanke Biological Technology Co., Ltd., Shenzhen, China), mouse anti-IL6ST antibody (ab27359, 1:400, Abcam), mouse anti-STAT3 antibody (ab119352, 1:300, Abcam), mouse anti-NF-kB p65 antibody (#6956, 1:500, Cell Signaling Technology (CST), Boston, MA, United States), mouse anti-(nuclear factor erythroid 2–related factor 2) Nrf2 antibody (ab89443, 1:300, Abcam), mouse anti-vimentin antibody (ab20346, 1:400, Abcam), mouse antiE-cadherin (ab238099, 1:400, Abcam), mouse anti-(alpha-smooth muscle actin) α-SMA (ab88979, 1:300, Abcam), and mouse anti-GAPDH antibody (ab9484, 1:300, Abcam). Signals were visualized by an enhanced chemiluminescence (ECL) kit (Habersham, Little Chalfont, United Kingdom). We used GAPDH as an endogenous control to normalize protein expression. The expression levels of the proteins were quantified by ImageJ software 1.8.0 (National Institutes of Health, Bethesda, MD, United States). The quantitative results were presented as the relative expression levels of target proteins normalized to the corresponding loading controls.

### Cell Viability Assay

The CCK-8 proliferation assay kit was used to determine the proliferation of cells. First, cells were incubated for 24 h, then 3 × 10^3^ cells were seeded into 96-well plates, and transfected with the indicated RNA duplexes. Then, the cells were treated with 10 μL of CCK-8 solution (WST-8, Dojindo Laboratories, Tokyo) for 0, 24, and 72 h, respectively, and the culture plate was incubated for an additional 2 h. The absorbance was measured at 450 nm by using a microplate reader (Bio-Rad, Hercules, CA, United States).

### Transwell Invasion Assay

The invasive ability of cells was determined by using an 8.0-µm 24-well Boyden chamber. The upper surface of the Transwell filter was coated with Matriel (BD, New Jersey, United States). First, the cells were planted into an 8.0-μm chamber plate, then 300 μl of serum-free DMEM was added to the upper compartment of the chamber, and 500 μl of DMEM supplemented with 10% FBS was added to the lower chamber for 48 h incubations. Then, the noninvasive cells on the upper side of the chamber were removed with a cotton swab, and the invasive cells were fixed in 4% paraformaldehyde and stained with 0.1% crystal violet solution. The Olympus IX70 inverted microscope (Olympus Corp, Tokyo, Japan) was used to observe the infiltrated cells, and the best six fields of view were randomly selected, and each experiment was repeated three times.

### Enzyme-Linked Immunosorbent Assay (ELISA)

HK-2 cells were transfected with MALAT1 siRNA and NC siRNA and then treated by NG or HG. The cells were ground and centrifuged, and the contents of IL-6 and TNF-α in the cell lysate were determined by ELISA kits (Thermo Fisher Scientific, Waltham, MA, United States) according to the manufacturer’s instructions.

### Luciferase Reporter Gene Assay

StarBase (http://starbase.sysu.edu.cn/index.php) was used to predict potential binding between lncRNA MALAT1 and miR-2355-3p. To further identify potential relationship, we performed luciferase reporter gene assay. The potential binding between miR-2355-3p and IL6ST was also predicted by StarBase and verified by luciferase reporter gene assay. First, we cloned the wild-type and mutant 3′-UTR sequences of lncRNA MALAT1 into the pGL3 promoter vector containing the luciferase reporter gene, respectively. The wild-type 3′-UTR of MALAT1 (containing the binding sites for miR-2355-3p) primer sequences were as follows: forward: 5′-TTA AAG TAG GAC AAC CAT GG-3′ and reverse: 5′-TTG CAG GCA AAT TAA TGG CC-3’. The mutant 3′-UTR of MALAT1 (six nucleotides were mutated in the binding sites) primer sequences were as follows: forward: 5′-TTG GGA TGG TCT TAA CAG GGA-3′ and reverse: 5′-GAA TTG GGA AGC TGG GGG AA-3′. The wild-type 3′-UTR of IL6ST (containing the binding sites for miR-2355–3p) primer sequences were as follows: forward: 5′-AAA​GGA​AGG​ACA​ATA​TAA​AG-3′, and reverse: 5′-AAA​ATT​GCA​GTG​AGC​CAG​CG-3’. The mutant 3′-UTR of IL6ST (six nucleotides were mutated in the binding sites) primer sequences were as follows: forward: 5′-TAA GCG ATT CTC CTA CCT TGG-3′ and reverse: 5′-AAG CTC ACT GTG ACC AGA GC-3’. HK-2 cells were seeded into 24-well plates and co-transfected with luciferase plasmid and wild-type lncRNA MALAT1 or mutant lncRNA MALAT1 by using Lipofectamine^™^ 3,000 (Invitrogen, Carlsbad, CA, United States) when grown to approximately 70% confluence. Twenty-four h after transfection, cells were harvested and analyzed by using a luciferase reporter gene kit (Promega, Madison, Wisconsin, United States) to normalize luciferase reporter activity to renilla luciferase activity.

### RNA Pull-Down Assay

To verify the relationship between lncRNA MALAT1 and miR-2355-3p, the full-length miR-2355-3p transcript was biotinylated. Then, biotin-labeled miR-2355-3p was synthesized and transfected into HEK293T cells. Biotin–miR-2355-3p probe was transcribed and purified by GenePharma Company (Shanghai, China) *via* the AmpliScribe^™^ T7-Flash^™^ Biotin-RNA Transcription Kit (Epicenter, Madison, Wisconsin, United States). After 48 h, the cells were washed and lysed, and then, the extract was incubated with avidin-anchored magnetic material at 4°C for 3 h. Then, the beads were washed twice with ice-cold buffer, three times with low-salt buffer, and once with high-salt buffer. Finally, the beads were washed with buffer, the RNA–RNA complex was eluted, and the RNA pull-down products were detected by RT-qPCR.

### Histopathological Examination of the Renal Tissues

Right sides of renal tissues from rats were removed at the end of experiment and fixed in 4% buffered paraformaldehyde at 4°C for 2 weeks. After fixed samples were dehydrated, embedded, and sliced, the slices were subjected to routine hematoxylin and eosin (H&E) staining (WLA051a, Wanlei-bio, China) to determine the histopathological changes in the kidney according to the manufacturer’s instruction. Finally, the sections were observed and photographed with an optical microscope (DP73, OLUMPUS, Japan).

Renal tissues were dehydrated, embedded in paraffin, sectioned 5 μm thick, and stained with Masson’s trichrome staining (K7298, IMEB inc. San Marcos, CA), periodic acid-Schiff (PAS) (BA-4114, Baso, China) and periodic acid silver-methenamine (PAM) staining (Shanghai zhuocai Biotechnology Co., Ltd., Shanghai, China). Each sample slice was observed under a microscope (DP73, OLUMPUS, Japan) at a magnification of 200× or 400×.

### Statistical Analysis

Statistical analyses were performed on SPSS 19.0 (IBM SPSS, Armonk, NY, United States). All quantitative results were presented as mean ± standard error of mean (SEM) of three independent experiments. The significance of the differences between two groups, unless for paired comparison which was noted specially, was conducted with Student’s *t*-test. Analysis of variance (ANOVA) was used to compare the significance among multiple groups, and multiple comparisons were performed by using Tukey-Kramer correction. *p* < 0.05 was considered to be statistically significant.

## Results

### Long Noncoding RNA Metastasis–Associated Lung Adenocarcinoma Transcript 1 Level Was Upregulated in Kidney Tissues of Streptozotocin-Induced Diabetic Rats and High Glucose–Treated HK-2 or 293T Cells

**FIGURE 1:**
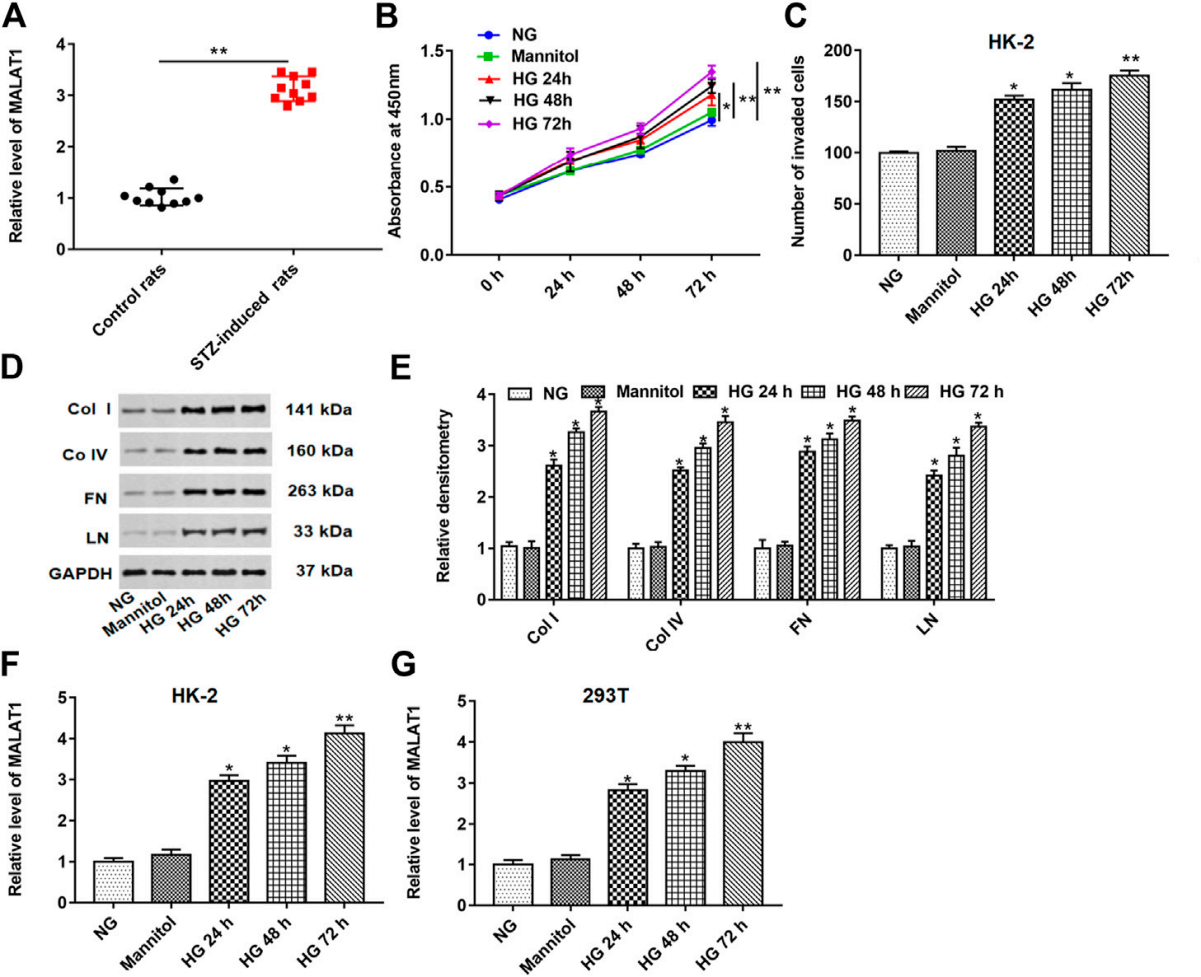
lncRNA MALAT1 was upregulated in STZ-induced diabetic rats and HG-treated HK-2 or 293T cells. **(A)** Expression of the MALAT1 level was detected by RT-qPCR in STZ-induced diabetic rats (n = 10) and control rats (n = 10). HK-2 cells were treated with NG, mannitol, and HG for 24, 48, and 72 h, respectively. **(B and C)** Effects of different treatments on cell proliferation and invasion of HK-2 cells at 0, 24, 48, and 72 h were detected by CCK-8 and Transwell assays. *p < 0.05 vs. NG or mannitol group, **p < 0.01 vs. NG or mannitol group. **(D and E)** Effects of different treatments on the expression of col I, col IV, FN, and LN in HK-2 cells were detected by densitometric analysis. *p < 0.05 or **p < 0.01 vs. NG group or mannitol group. **(F and G)** lncRNA MALAT1 expression was detected by RT-qPCR in HK-2 or 293T cells. *p < 0.05 or **p < 0.01 vs. NG or mannitol group.

As shown in Figure 1A, the MALAT1 level was upregulated in STZ-induced diabetic rat (n = 10) tissues compared with control rats (n = 10). HK-2 cells were treated with normal glucose (NG), mannitol, HG for 24, 48, and 72 h. HG treatment significantly enhanced cell proliferation and invasion and upregulated fibrosis–related protein (col I, col IV, FN and, LN) levels in a time-dependent manner compared with the NG or mannitol group (Figures 1B–E). Furthermore, we demonstrated that the MALAT1 level was upregulated in a time-dependent manner in HG-treated HK-2 and 293T cells (Figures 1F,G). These data indicate that HG treatment could aggravate lncRNA MALAT1 expression *in vivo* and *in vitro*.

### Knockdown of Long Noncoding RNA Metastasis–Associated Lung Adenocarcinoma Transcript 1 Suppressed the Expression of Collagen I, Collagen IV, Fibronectin, and Laminin in High Glucose–Treated HK-2 Cells

**FIGURE 2:**
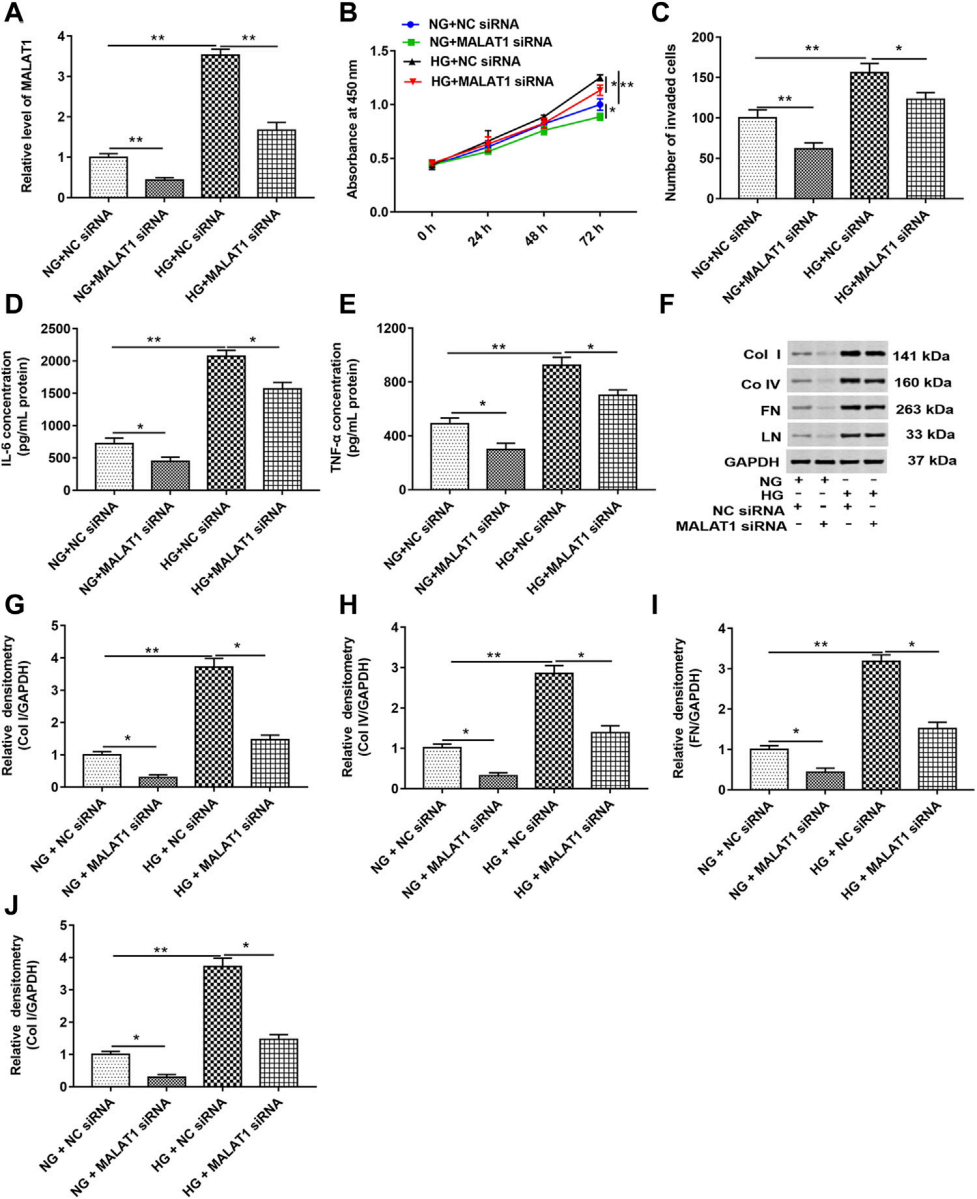
Knockdown of lncRNA MALAT1 downregulated col I, col IV, FN, and LN protein levels and inhibited cell proliferation and invasion in HG-treated HK-2 cells. MALAT1 siRNA and NC siRNA were transfected into cells and then treated with NG and HG, respectively: **(A)** RT-qPCR was used to determine the expression of MALAT1 in cells. **p < 0.01 vs. NG + NC siRNA group or HG + NC siRNA group. CCK-8 **(B)** and Transwell assays **(C)** were used to detect the effects of these two vectors on cell proliferation and invasion. *p < 0.05 vs. NG + NC siRNA group or HG + NC siRNA group, **p < 0.01 vs. NG + NC siRNA group or HG + NC siRNA group. **(D and E)** ELISA was used to determine the contents of IL-6 and TNF-α in cell lysate when these two vectors were transfected into NG- or HG-treated cells. *p < 0.05 vs. NG + NC siRNA group or HG + NC siRNA group, **p < 0.01 vs. NG + NC siRNA group. **(F)** Western blotting was used to detect the expression of col Ⅰ, col Ⅳ, FN, and LN in NG- or HG-treated cells after transfection with these two vectors. The histogram in **(G–J)** shows the densitometric analysis of the blots (col I, col IV, FN, and LN) normalized to GAPDH. *p < 0.05 vs. NG + NC siRNA group or HG + NC siRNA group, **p < 0.01 vs. NG + NC siRNA group.

HK-2 cells were transfected with MALAT1 siRNA and NC siRNA and then treated with HG (72 h) or NG, respectively. RT-qPCR, CCK-8, and Transwell assays showed that HG treatment upregulated the MALAT1 level and enhanced cell proliferation and invasion. Knockdown of MALAT1 downregulated the MALAT1 level and suppressed cell proliferation and invasion in untreated or treated cells (Figures 2A–C). As shown in Figures 2D,E, HG treatment significantly increased the concentrations of IL-6 and TNF-α in cell lysate compared with NG-treated cells. Knockdown of lncRNA MALAT1 significantly decreased the concentrations of IL-6 and TNF-α in NG- or HG-treated cell lysate. In addition, the data of Western blotting showed that col I, col IV, FN, and LN protein levels were upregulated in HG-treated cells, while lncRNA MALAT1 knockdown significantly downregulated col I, col IV, FN, and LN protein levels in NG- or HG-treated cells (Figures 2F–J). These results suggest that lncRNA MALAT1 may upregulate col I, col IV, FN, and LN protein levels in DN.

### Overexpression of Long Noncoding RNA Metastasis–Associated Lung Adenocarcinoma Transcript 1 Upregulated Collagen I, Collagen IV, Fibronectin, and Laminin Protein Levels in High Glucose–Treated HK-2 Cells

**FIGURE 3:**
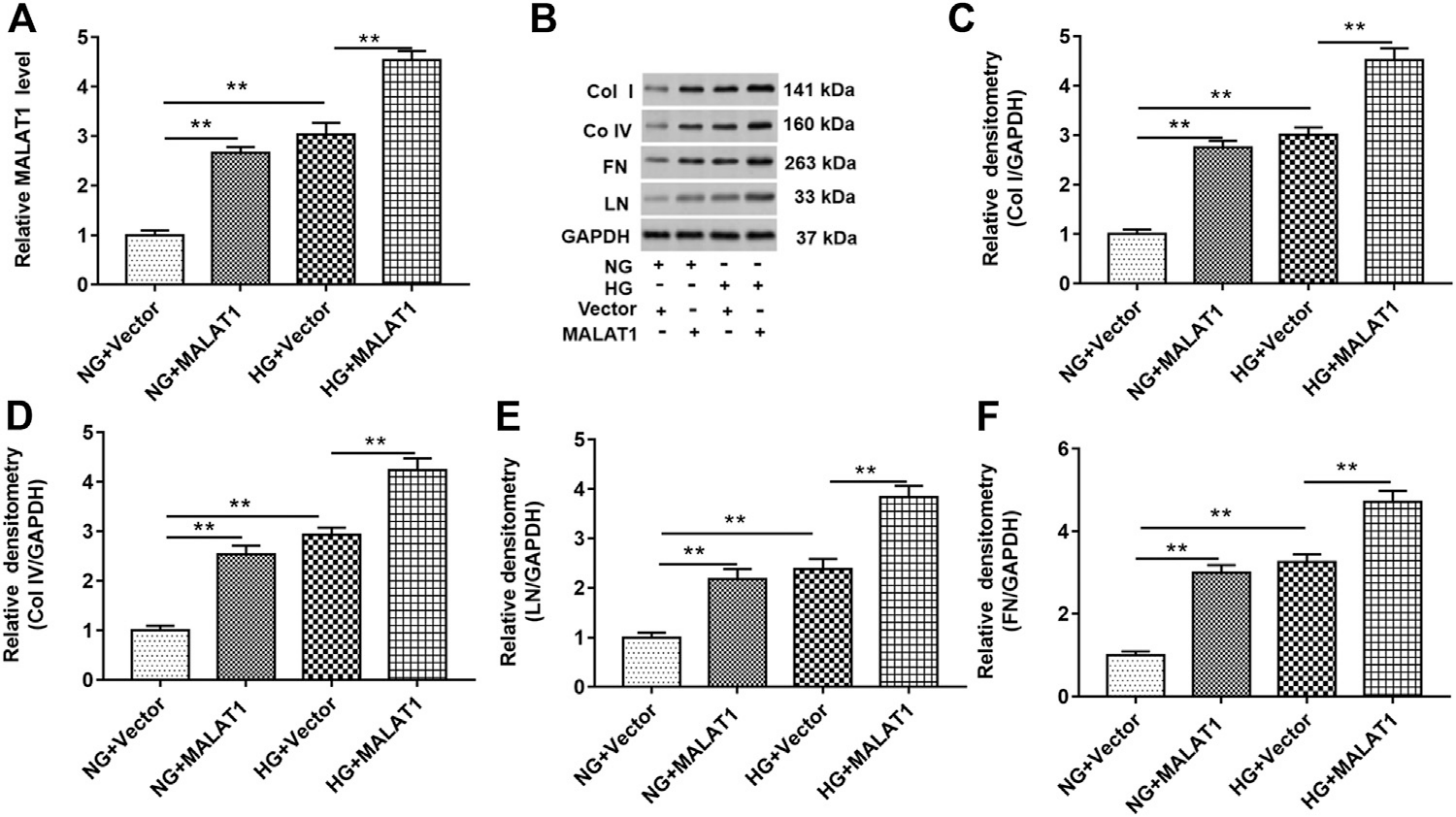
Overexpression of lncRNA MALAT1 upregulated col I, col IV, FN, and LN protein levels in HG-treated HK-2 cells. Cells were transfected with pcDNA-MALAT1 (MALAT1) and pcDNA3.1 (vector) and then treated with NG or HG, respectively: **(A)** RT-qPCR was used to determine the expression of MALAT1 in cells transfected with these two vectors. **p < 0.01 vs. NG + vector group or HG + vector group. **(B)** Expression of col I, col IV, FN, and LN in cells transfected with these two vectors was detected by Western blotting. The histogram in **(C–F)** shows the densitometric analysis of the bands (col I, col IV, FN, and LN) normalized to GAPDH. **p < 0.01 vs. NG + vector group or HG + vector group.

HK-2 cells were treated with NG or HG treatment after pcDNA-lncMALAT1 and pcDNA3.1 (a negative control of pcDNA-lncMALAT1) were transfected into cells, respectively. The transfection efficiency of pcDNA3.1 vector into HK-2 cells was about 80%. As shown in Figure 3A, overexpression of MALAT1 upregulated the MALAT1 level in NG-treated cells. HG treatment significantly upregulated the expression of lncRNA MALAT1, and lncRNA MALAT1 overexpression also upregulated MALAT1 levels in HG-treated cells. As shown in Figures 3B–F, col I, col IV, FN, and LN protein levels were upregulated in NG-treated cells transfected with pcDNA-lncMALAT1. Overexpression of MALAT1 increased these protein levels in HG-treated cells, compared with HG-treated cells transfected with vectors.

### Long Noncoding RNA Metastasis–Associated Lung Adenocarcinoma Transcript 1 Directly Bound to miR-2355-3p, and Overexpression of miR-2355-3p Downregulated Collagen I, Collagen IV, Fibronectin, and Laminin Protein Levels in High Glucose–Treated HK-2 Cells

**FIGURE 4:**
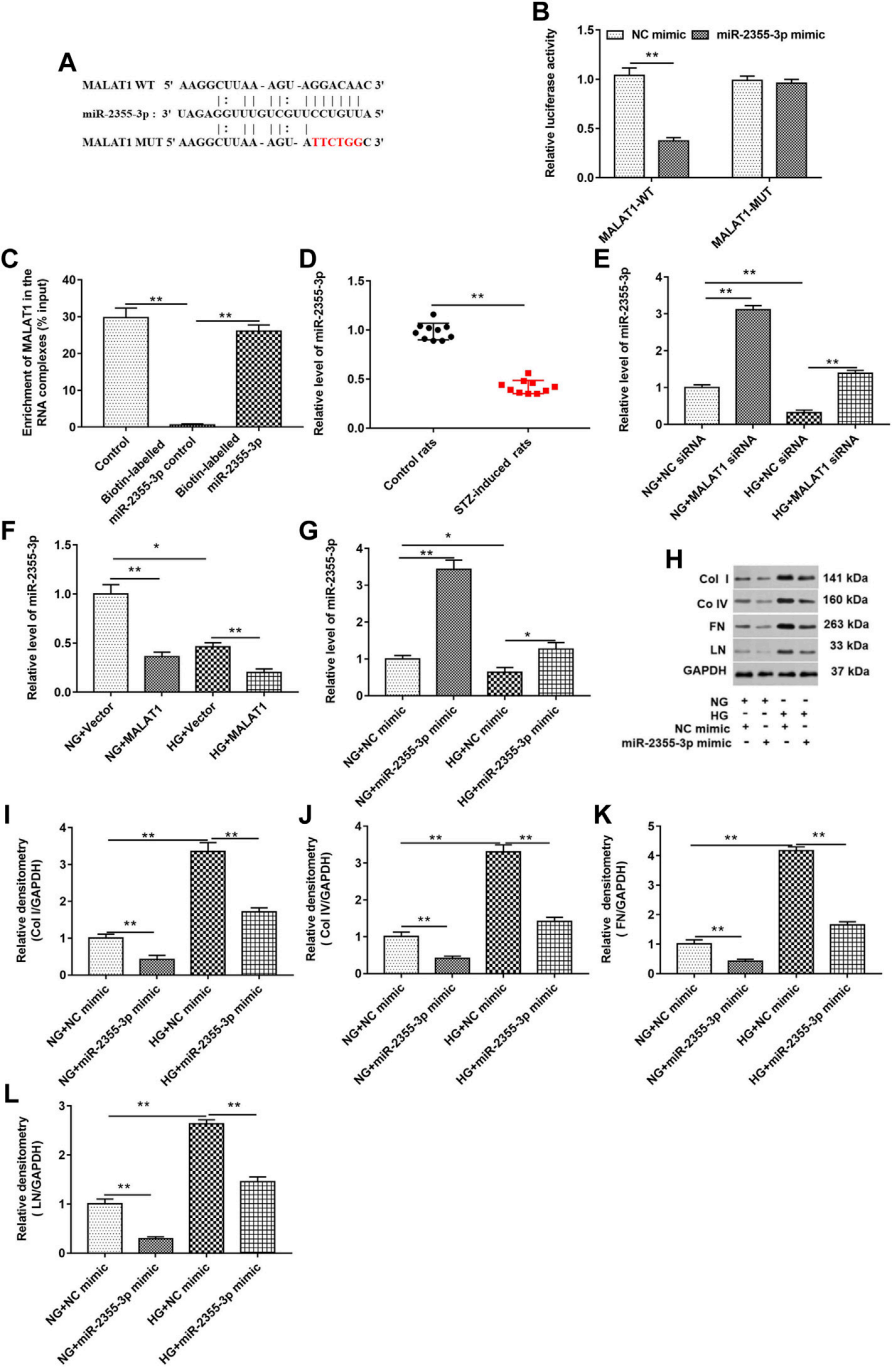
lncRNA MALAT1 directly bound to miR-2355-3p, and overexpression of miR-2355-3p downregulated col I, col IV, FN, and LN protein levels in HG-treated HK-2 cells. **(A)** StarBase (http://starbase.sysu.edu.cn/) was used to predict the binding between MALAT1 and miR-2355-3p. **(B)** Luciferase reporter gene assay was used to verify the binding between MALAT1 and miR-2355-3p. **p < 0.01 vs. NC mimic group. **(C)** Pull-down assay was used to detect the binding ability of miR-2355-3p and lncRNA MALAT1. **p < 0.01 vs. control or biotin-labeled miR-2355-3p group. **(D)** miR-2355-3p level in STZ-induced diabetic rats (n = 10) and control rats (n = 10) was detected by RT-qPCR. **p < 0.01 vs. control rats. **(E and F)** miR-2355-3p level was measured with RT-qPCR in NG- or HG-treated cells transfected with MALAT1 siRNA, NC siRNA, pcDNA-MALAT1, or pcDNA3.1. *p < 0.05 vs. NG + vector group, **p < 0.01 vs. NG + NC siRNA group, HG + NC siRNA group, NG + vector group, or HG + vector group. **(G)** miR-2355-3p level was measured with Western blot assay in NG- or HG-treated cells transfected with miR-2355-3p mimic or NC mimic. *p < 0.05 vs. NG + NC mimic, **p < 0.01 vs. NG + NC mimic group or HG + NC mimic group. **(H)** Expression of col I, col IV, FN, and LN in NG- or HG-treated cells transfected with miR-2355-3p mimic or NC mimic was detected by Western blotting. The histogram in **(I–L)** presents the densitometric analysis of the blots (col I, col IV, FN, and LN) normalized to GAPDH. **p < 0.01 vs. NG + NC mimic or HG + NC mimic group.

As shown in Figures 4A,B, StarBase (http://starbase.sysu.edu.cn/index.php) and luciferase reporter gene assay were used to predict and verify the binding of lncRNA MALAT1 and miR-2355-3p. The results showed that wild-type lncRNA MALAT1 could target the 3′UTRs of miR-2355-3p (5′-UUGUCCU-3′). The relationship between the targeted binding of lncRNA MALAT1 and miR-2355-3p was further confirmed by pull-down assay (Figure 4C). lncRNA MALAT1 could pull down miR-2355-3p labeled with the miR-2355-3p probe in cell lysate. The miR-2355-3p level was downregulated in STZ-induced diabetic rats (n = 10) tissues compared with control rats (n = 10) (Figure 4D). HG treatment downregulated the miR-2355-3p level, and knockdown of MALAT1 upregulated the miR-2355-3p level in HG-untreated and -treated cells (Figure 4E), while overexpression of lncRNA MALAT1 significantly inhibited the expression of miR-2355-3p in HG-untreated and -treated cells (Figure 4F). Overexpression of miR-2355-3p significantly increased the expression of miR-2355-3p in NG-treated cells. However, overexpression of miR-2355-3p rescued the inhibitory effect of HG-treated cells on miR-2355-3p (Figure 4G). miR-2355-3p overexpression upregulated col I, col IV, FN, and LN protein levels in NG-treated cells, while it downregulated these four protein levels in HG-treated cells (Figures 4H–L). These data suggest that MALAT1 could sponge miR-2355-3p to regulate DN progression.

### IL6ST Bound to MALAT1

**FIGURE 5:**
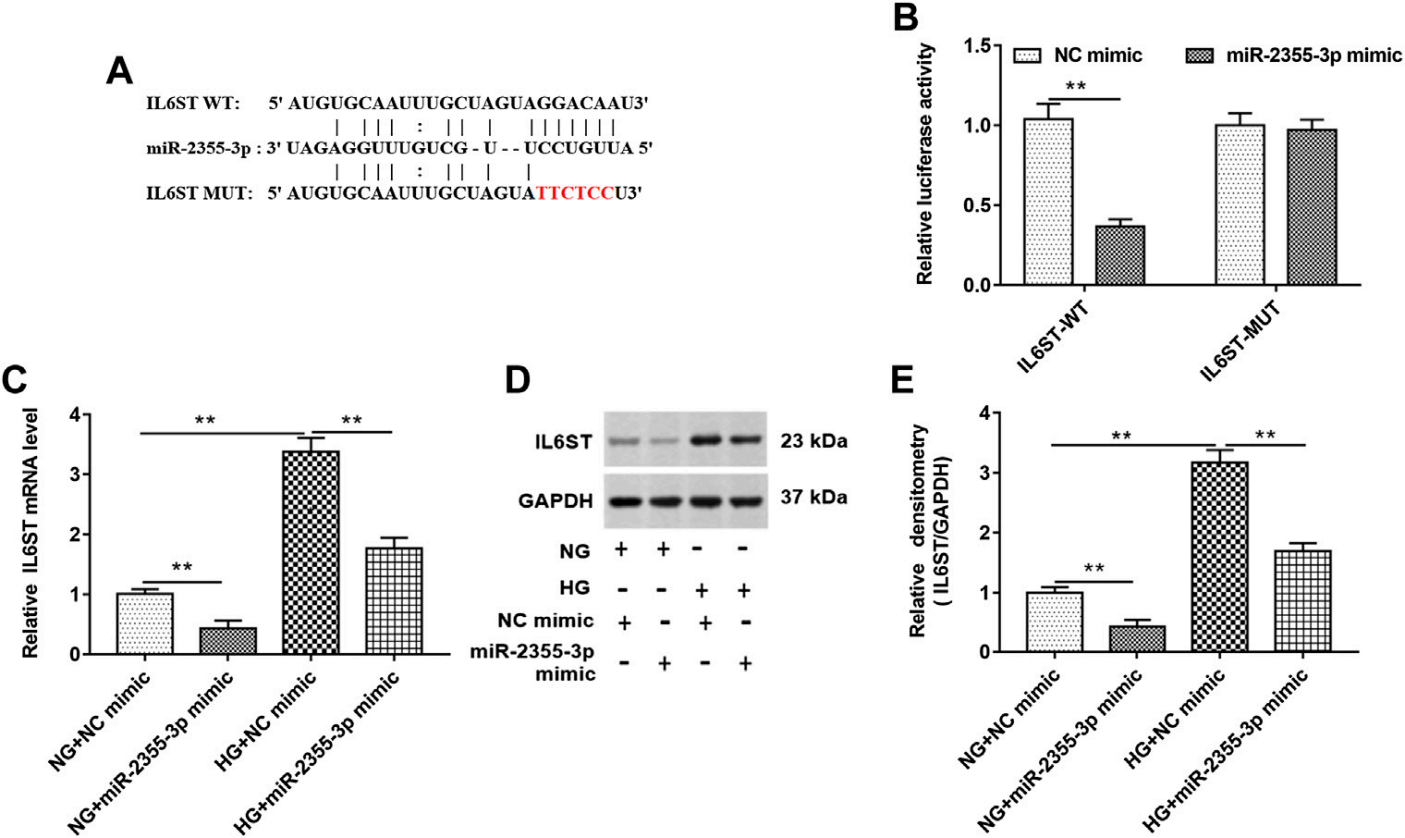
IL6ST was a target of miR-2355-3p. **(A)** StarBase (http://starbase.sysu.edu.cn/) was used to predict the binding between IL6ST and miR-2355-3p. **(B)** Luciferase reporter gene assay in HEK293T cells was used to verify the binding between IL6ST and miR-2355-3p. **p < 0.01 vs. NC mimic group. **(C and D)** The expression of IL6ST was detected by RT-qPCR and Western blotting in NG- or HG-treated cells transfected with miR-2355-3p mimic or NC mimic. **p < 0.01 vs. NG + NC mimic group or HG + NC mimic group. The histogram in **(E)** presents the densitometric analysis of the blots (IL6ST) normalized to GAPDH. **p < 0.01 vs. NG + NC mimic group or HG + NC mimic group.

StarBase (http://starbase.sysu.edu.cn/) and luciferase reporter gene assay were used to predict and verify the binding between IL6ST and miR-2355-3p (Figures 5A,B). As shown in 5B, luciferase reporter gene assay showed that wild-type IL6ST could bind to miR-2355-3p. In addition, RT-qPCR and Western blotting assays showed that overexpression of miR-2355-3p significantly decreased the IL6ST mRNA and protein level in NG-treated HK-2 cells. HG treatment significantly increased the expression of IL6ST mRNA and protein, and overexpression of miR-2355-3p significantly suppressed the expression of IL6ST mRNA and protein in HG-treated cells (Figures 5C–E). These results confirm that miR-2355-3p plays an inhibitory role by inhibiting IL6ST in DN progression.

### Effects of IL6ST Mediated NF-kB Signal Transduction on Collagen I, Collagen IV, Fibronectin, and Laminin Protein Levels in High Glucose–Induced HK-2 Cells

**FIGURE 6:**
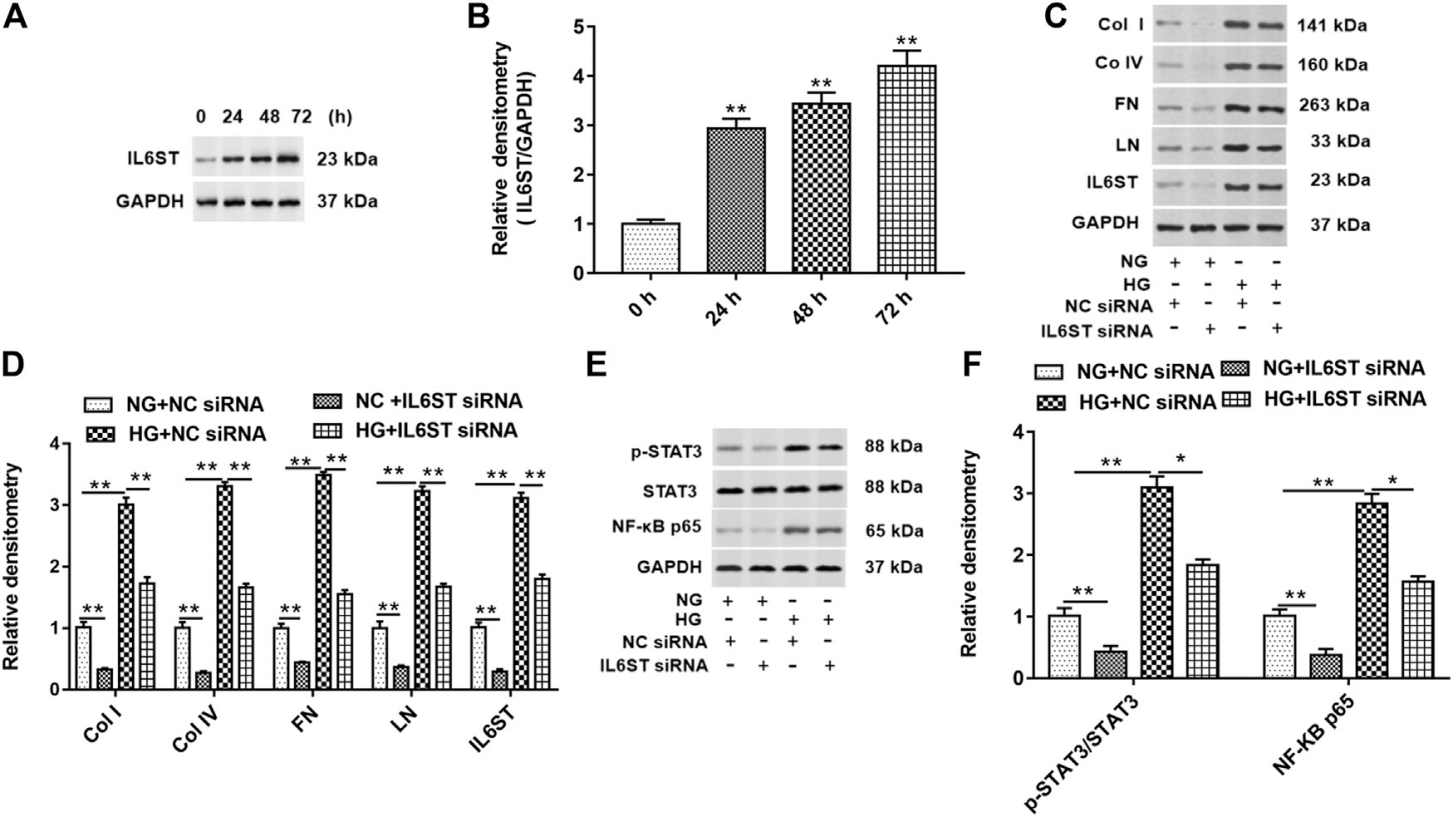
IL6ST knockdown downregulated col I, col IV, FN, and LN protein levels and blocked the STAT3/NF-kB signaling pathway in HG-treated HK-2 cells. **(A)** Western blotting was used to detect the IL6ST level in cells treated by HG for 0, 24, 48, and 72 h. The histogram in **(B)** shows the densitometric analysis of the blots (IL6ST) normalized to GAPDH. **p < 0.01 vs. 0 h group. **(C)** col I, col IV, FN, LN, and IL6ST protein levels in cells transfected with IL6ST siRNA or NC siRNA were detected by Western blotting. **(D)** Densitometric analysis of the blots (col I, col IV, FN, LN, and IL6ST) normalized to GAPDH. **p < 0.01 vs. NG + NC siRNA or HG + NC siRNA group. **(E)** Western blotting was used to detect the STAT3 phosphorylation level, total STAT3, and NF-kB p65 protein levels in NG- or HG-treated cells. The histogram in **(F)** presents the densitometric analysis of the blots (p-STAT3/STAT3 and NF-kB p65) normalized to GAPDH. **p < 0.01 vs. NG + NC siRNA group or HG + NC siRNA group.

HG treatment significantly increased the expression of IL6ST in cells at 24, 48, and 72 h (Figures 6A,B). IL6ST siRNA and NC siRNA were transfected into cells and treated with HG or NG. As shown in Figures 6C,D, knockdown of IL6ST significantly decreased the expression of col I, col IV, FN, LN, and IL6ST proteins in NG-treated cells. On the contrary, HG treatment promoted the expression of these five proteins. In addition, IL6ST knockdown reversed the upregulation of HG treatment on these five proteins. Moreover, knockdown of IL6ST significantly reduced the STAT3 phosphorylation level and NF-kB p65 protein expression in NG-treated cells but did not affect the total STAT3 expression. However, HG treatment significantly upregulated the STAT3 phosphorylation level and NF-kB p65 expression in cells. IL6ST knockdown significantly reversed the increase of the STAT3 phosphorylation level and NF-kB p65 protein expression caused by HG treatment (Figures 6E,F). These results suggest that IL6ST may be a key regulatory factor in the NF-kB signaling pathway in DN progression.

### miR-2355-3p Knockdown Reversed the Inhibitory Effect of Metastasis-Associated Lung Adenocarcinoma Transcript 1 Knockdown on the Expression of NF-kB Pathway–Related Genes in High Glucose–Treated HK-2 Cells

**FIGURE 7:**
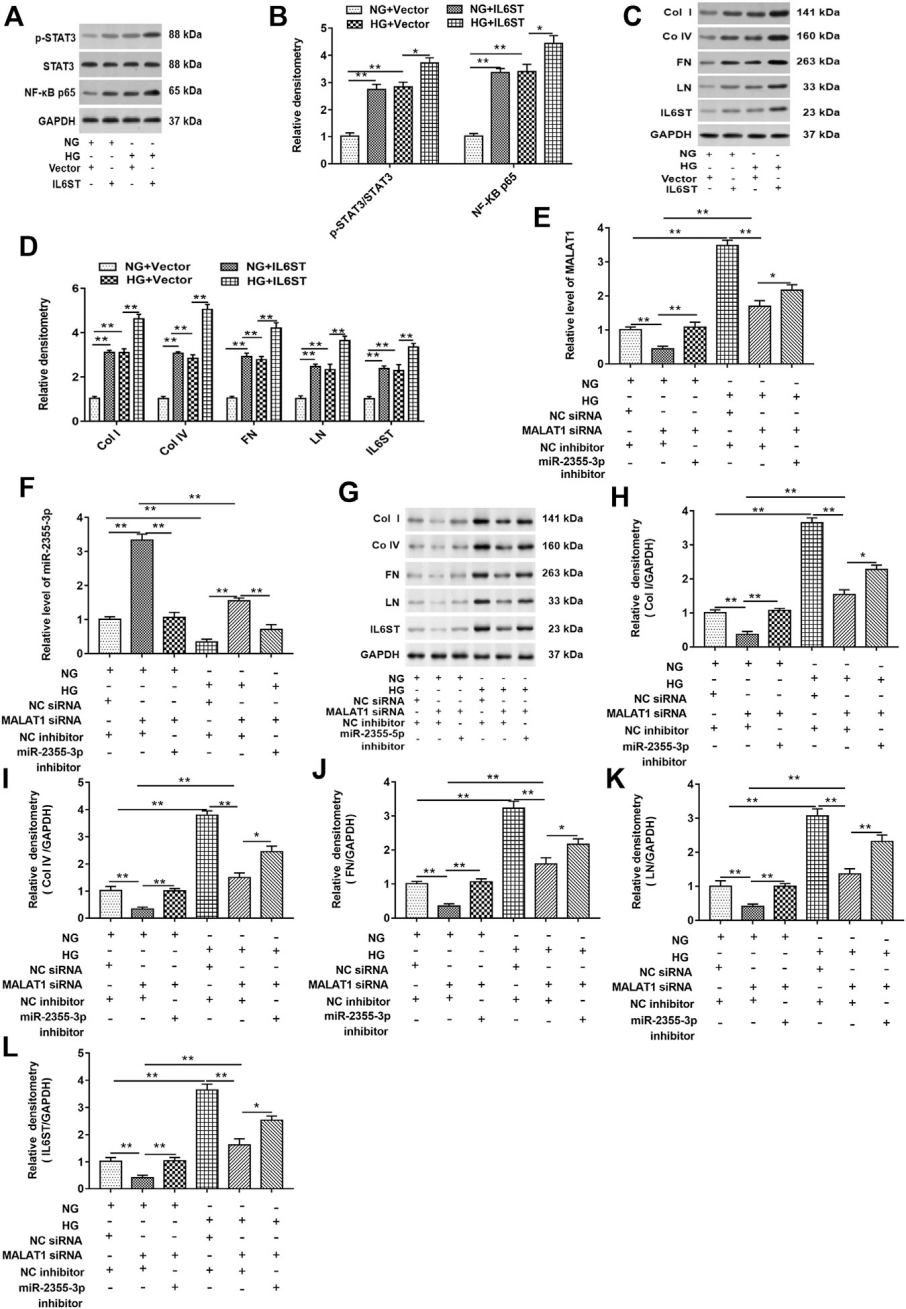
miR-2355-3p knockdown reversed the inhibitory effect of lncRNA MALAT1 knockdown on col I, col IV, FN, LN, and IL6ST protein levels in HG-treated HK-2 cells. Cells were transfected with pcDNA-IL6ST (IL6ST) or pcDNA3.1 (vector) and then treated by NG or HG, respectively: **(A)** Western blotting was used to detect the STAT3 phosphorylation level, total STAT3, and NF-kB p65 protein levels in cells transfected with these two vectors. **(B)** Densitometric analysis of the blots (p-STAT3/STAT3 and NF-kB p65) normalized to GAPDH. **p < 0.01 vs. NG + vector group or HG + vector group, *p < 0.05 vs. HG + vector group. **(C)** col I, col IV, FN, LN, and IL6ST protein levels were measured by Western blotting in cells transfected with these two vectors. The histogram in **(D)** shows the densitometric analysis of the blots (col I, col IV, FN, LN, and IL6ST) normalized to GAPDH. **p < 0.01 vs. NG + vector group or HG + vector group. Cells were transfected with MALAT1 siRNA together with the NC inhibitor or the miR-2355-3p inhibitor and NC siRNA together with the NC inhibitor and then treated by NG or HG, respectively: **(E and F)** RT-qPCR was used to examine the expression of MALAT1 and miR-2355-3p in cells. **(G)** The expression of col I, col IV, FN, LN, and IL6ST in NG- or HG-treated cells was detected by Western blotting. The histogram in **(H–L)** presents the densitometric analysis of the blots (col I, col IV, FN, LN, and IL6ST) normalized to GAPDH. **p < 0.01 vs. NG + NC siRNA + NC inhibitor group, NG + MALAT1 siRNA + NC inhibitor group, or HG + NC siRNA + NC inhibitor group, *p < 0.05 vs. HG + MALAT1 siRNA + NC inhibitor group.

First, cells were transfected with blank vector and pcDNA-IL6ST and then treated with HG or NG, respectively. As shown in Figures 7A,B, in cells with or without HG treatment, IL6ST overexpression upregulated the STAT3 phosphorylation level and NF-kB p65 protein expression in cells but did not affect the total STAT3 expression. Moreover, overexpression of IL6ST significantly increased the STAT3 phosphorylation level and NF-kB p65 protein expression in HG-treated cells. The IL-6/STAT3 signaling pathway plays an important role in tissue damage and is accompanied by tissue fibrosis (Ranganathan et al., 2013; Xiao et al., 2014). And IL-6 responses are mediated *via* IL6ST-STAT3–dependent mechanisms in colorectal cancer (Ahmad et al., 2017). Overexpression of IL6ST significantly upregulated the protein levels of col I, col IV, FN, LN, and IL6ST in NG-treated cells, and these protein levels were also upregulated in HG-treated cells. Moreover, IL6ST overexpression upregulated IL6ST, col I, col IV, FN, and LN levels in HG-treated cells (Figures 7C,D). Furthermore, knockdown of lncRNA MALAT1 significantly inhibited lncRNA MALAT1 expression, and the protein levels of col I, col IV, FN, LN, and IL6ST, while promoted miR-2355-3p expression. However, miR-2355-3p inhibitor reversed the inhibitory effects of MALAT1 silence on MALAT1, col I, col IV, FN, LN, and IL6ST protein expression and the promotion effect on miR-2355-3p expression (Figure 7E–L).

### Streptozotocin-Induced Diabetic Rats Aggravated Renal Injury

**FIGURE 8:**
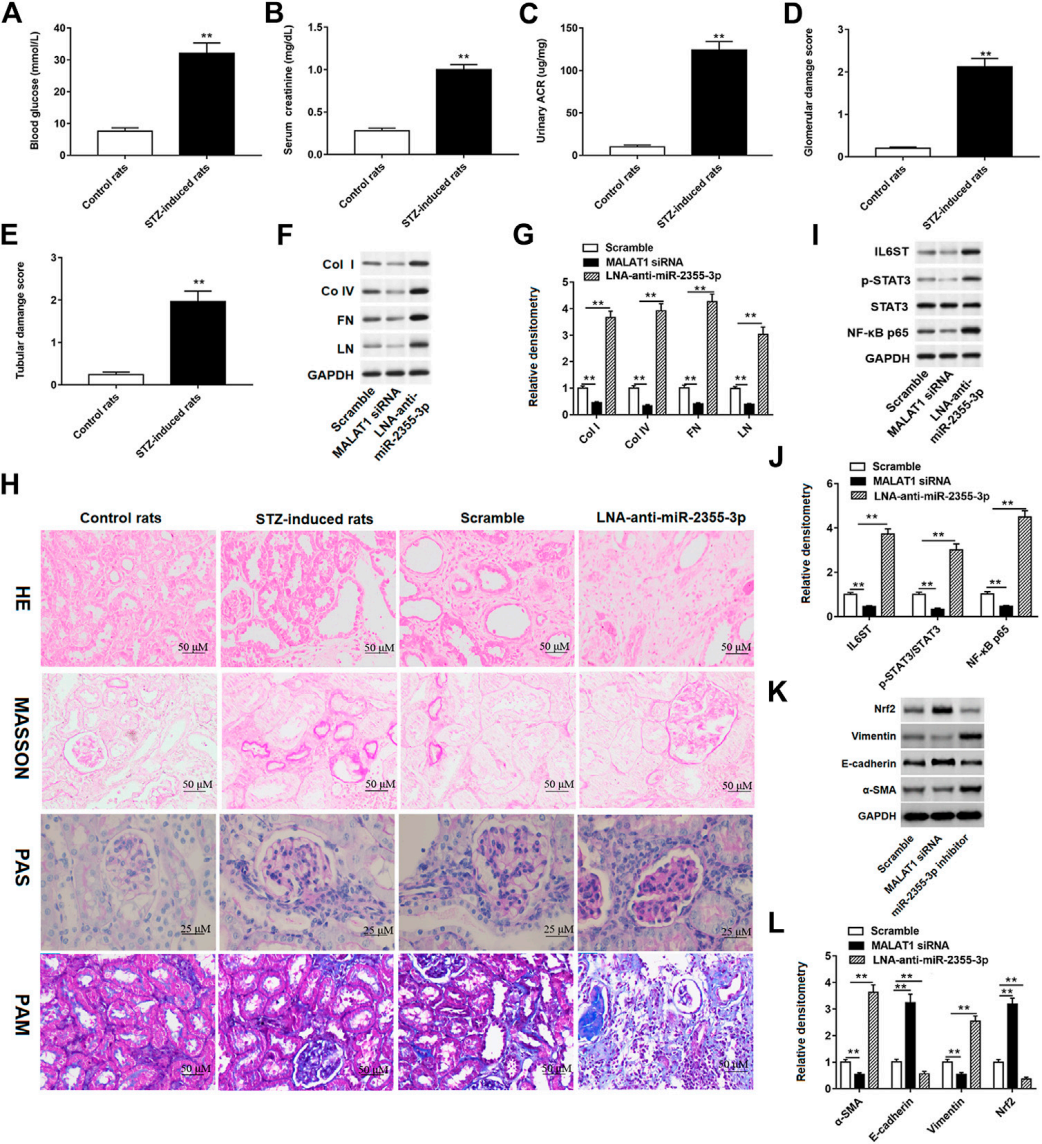
STZ-induced diabetic rats aggravated renal injury and promoted renal fibrosis. Contents of **(A)** blood glucose, **(B)** serum creatinine, and **(C)** urinary ACR were detected by ELISA in STZ-induced diabetic rats (n = 10) and control rats (n = 10). **p < 0.01 vs. control rats. **(D and E)** Glomerular and tubular damage scores were evaluated by the experimenter. **p < 0.01 vs. control rats. **(F)** Western blot assay was used to detect the col I, col IV, FN, and LN protein levels in STZ-induced diabetic rats administrated with MALAT1 siRNA or miR-2355-3p inhibitor. The histogram in **(G)** shows the densitometric analysis of the blots (col I, col IV, FN, and LN) normalized to GAPDH. **p < 0.01 vs. scramble group. **(H)** Renal tissues of rats were collected for H&E (×200, scale bar = 50 µm), Masson’s trichrome (×200, scale bar = 50 µm), PAS (×400, scale bar = 25 µm), and PAM staining (×200, scale bar = 50 µm). miR-2355-3p levels were measured in heart, liver, bladder, kidney and peritoneal tissues of STZ-induced diabetic rats administrated with LNA-anti-miR-2355-3p and scramble (Supplementary Figure S1). **p < 0.01 vs. scramble group. **(I)** Western blot assay was used to measure the IL6ST, p-STAT3, total STAT3, and NF-kB p65 protein levels in STZ-induced diabetic rats when rats were administrated with MALAT1 siRNA or LNA-anti-miR-2355-3p. The histogram in **(J)** presents the densitometric analysis of the blots (IL6ST, p-STAT3/STAT3, and NF-kB p65) normalized to GAPDH. **p < 0.01 vs. scramble group. **(K)** Several EMT marker protein levels, including Nrf2, vimentin, E-cadherin, and α-SMA, were detected by Western blotting in MALAT1 knockdown or antagomir-treated kidneys. The histogram in **(L)** shows the densitometric analysis of the blots (α-SMA, E-cadherin, vimentin, and Nrf2) normalized to GAPDH. **p < 0.01 vs. scramble group.

We conducted experiments in rats to verify the effects of MALAT1 and miR-2355-3p on STZ-induced diabetic rat blood glucose and renal function. These results showed that glucose levels in the blood (Figure 8A), creatinine in the plasma (Figure 8B), and the ACR (Figure 8C) were significantly increased in STZ-induced diabetic rats. In addition, STZ-induced lesions in renal tubules and collecting tubules were aggravated in diabetic rats (Figures 8D,E). Furthermore, MALAT1 siRNA and LNA-anti-miR-2355-3p were injected into rats, respectively, and the results showed that knockdown of MALAT1 inhibited the protein expression of col I, col IV, FN, and LN in rat renal tissues, while miR-2355-3p knockdown enhanced the protein expression of col I, col IV, FN, and LN (Figures 8F,G). H&E staining showed the obvious tubular epithelial disruption, hypertrophy of glomeruli, and renal fibrosis in STZ-induced diabetic rats, which were aggravated by administration of LNA-anti-miR-2355-3p compared with administration with scramble (a negative of LNA-anti-miR-2355-3p). Periodic acid–Schiff (PAS) staining showed mesangial expansion and increased glomerular size, Masson trichrome staining showed increased glomerular fibrosis and enlarged interstitial space, and PAM staining revealed glomerular atrophy, epithelial detachment, and glomerular basement membrane thickening in STZ-induced diabetic rats compared with control rats, and these characteristics were aggravated in diabetic rats administrated with LNA-anti-miR-2355-3p (Figure 8H).

**FIGURE 9:**
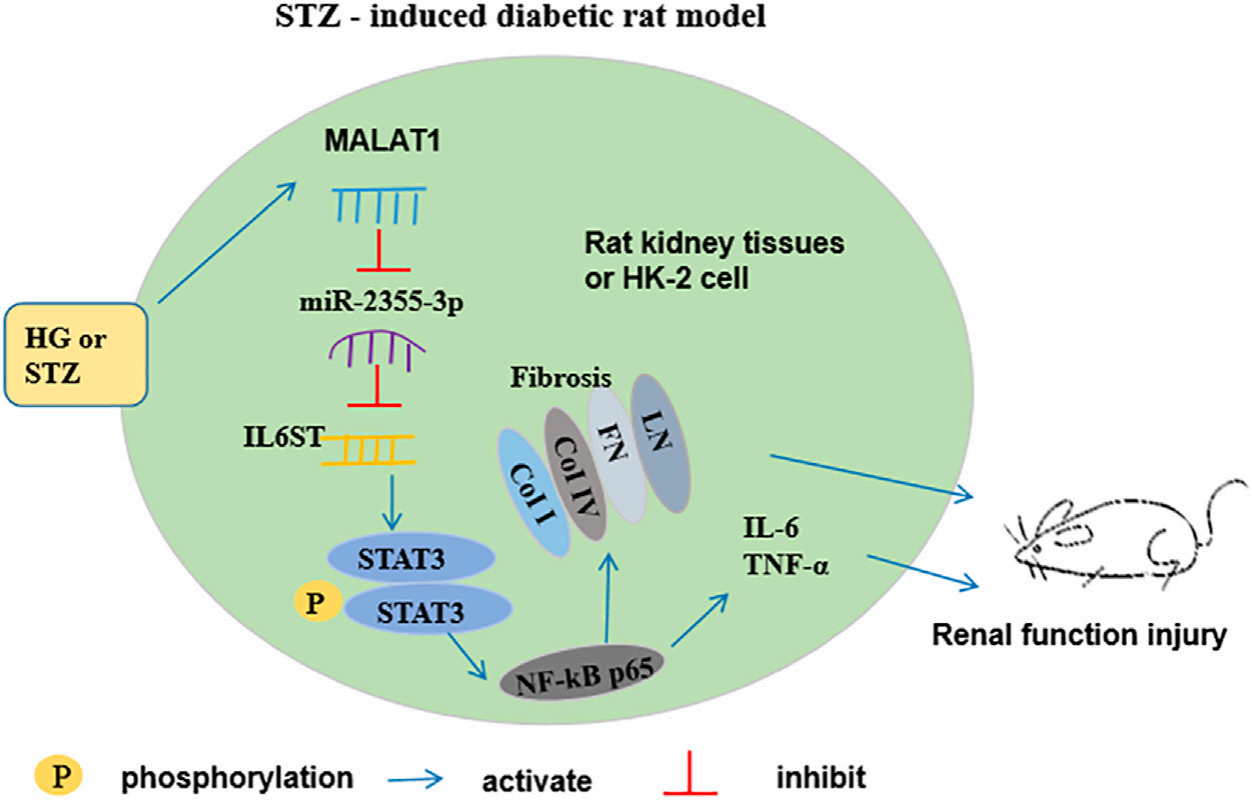
Schematic diagram for the functions and mechanism of lncRNA MALAT1 in regulating renal fibrosis in diabetic rats. MALAT1 is induced by high glucose in renal tubular epithelial cells *in vitro* and in renal tubular epithelium in STZ-diabetic rats *in vivo*, which sponges miR-2355-3p and positively regulates expression of IL6ST gene. IL6ST is an identified regulator to phosphorylate STAT3 and activates its downstream master pro-inflammatory transcription factor NF-kB. Then, NF-kB transcriptionally upregulates the fibrogenic marker genes, including LN, FN, coi I, and col IV, as well as secretion of pro-inflammatory cytokines IL-6 and TNF-α, thus promoting renal fibrosis in diabetic rats.

miR-2355-3p levels were downregulated in kidney and peritoneal tissues of STZ-induced diabetic rats administrated with LNA-anti-miR-2355-3p and scramble, while there was no significant change of miR-2355-3p levels in heart, liver, and bladder tissues (Supplementary Figure S1). Moreover, we demonstrated in rats that MALAT1 knockdown blocked the IL6ST/STAT3/NF-kB signaling pathway, while knockdown of miR-2355-3p activated this signaling pathway (Figures 8I,J). Results of Western blot assay demonstrated that the protein levels of EMT marker proteins α-SMA and vimentin were downregulated, and Nrf2 and E-cadherin protein levels were upregulated in renal tissues of MALAT1 knockdown diabetic rats. And Nrf2 and E-cadherin protein levels were downregulated, and α-SMA and vimentin protein levels were upregulated in renal tissues of diabetic rats administrated with miR-2355-3p inhibitor (Figures 8K,L). A cartoon figure showing the whole pathway is described in this article (Figure 9): lncRNA MALAT1, induced by HG in rat renal tubular epithelial cells, sponging miR-2355-3p to upregulate expression of IL6ST that can activate the STAT3/NF-kB pro-inflammatory and the fibrogenic axis, promotes renal fibrosis in rats with DN.

## Discussion

Our results determined that lncRNA MALAT1 was upregulated in HK-2 or 293T cell lines, and knockdown of MALAT1 inhibited HG-treated cell damage in HK-2 cells. In addition, MALAT1 directly bound to miR-2355-3p, and IL6ST was a target of miR-2355-3p. Our results confirmed that MALAT1 promotes HG-treated fibrosis by adsorbing miR-2355-3p on IL6ST *via* regulating the STAT3/NF-kB axis.

Higher levels of integrin β1 interact with DPP-4, affecting the TGF-β signal and activating the pro-endothelial-to-mesenchymal transition (EndMT) signal (Gupta and Sen, 2019; Srivastava et al., 2020a). In STZ-induced mice, SGLT2 knockout inhibited epithelial-mesenchymal transition (EMT) and restored all abnormal histology and function of the kidney. Inhibition of SGLT2 in renal tubular cells also inhibited the mesenchymal transition of adjacent endothelial cells (Li et al., 2020). In HK-2 cells, SGLT2 inhibitor decreased the abnormal expression of STAT1 and reversed the downregulation of E-cadherin and upregulation of α-SMA induced by HG (Huang et al., 2019). AcSDKP is a key and essential peptide to regulate the anti-fibrotic cross talk between miR-29s and miR-let-7s in endothelial cells. And this anti-fibrotic microRNA cross talk is critical to its anti-EndMT effect (Kanasaki et al., 2014; Srivastava et al., 2016; Li et al., 2017b). Moreover, ACEi upregulated anti-fibrinolytic microRNAs (miR-29 and miR-let-7 family members) and restored the anti-fibrotic cross talk of cultured endothelial cells, while ARB had the least effect, indicating that ACEi had an important anti-EndMT effect (Srivastava et al., 2020b). Our result revealed that administration of LNA-anti-miR-2355-3p enhanced the EMT effect in diabetic rats by upregulating α-SMA and vimentin levels and downregulating Nrf2 and E-cadherin protein levels.

When diabetes occurs, kidney function usually changes, such as glomerular filtration, increased renal blood flow, renal hypertrophy, and renal fibrosis (Bell et al., 2006; Deng et al., 2015; Amanzadeh et al., 2018). Some literature studies reported that diabetic mice (*db/db*) developed progressive proteinuria, glomerular mesangial matrix dilatation, glomerulosclerosis, mesangial dilation, and increased type IV collagen and fibronectin protein levels in the kidney (Tang et al., 2017; Wu et al., 2018). Similarly, *in vitro* and *in vivo*, our results also showed that HK-2 cells treated with HG or diabetic rats induced by STZ increased the protein levels of fibrosis genes including coi I, coi IV, FN, and LN, and renal tubular epithelial destruction, glomerular hypertrophy, mesangial expansion, and renal fibrosis were observed in diabetic rats.

Many literature studies reported that epigenetic factors regulate the occurrence and development of DN (Duan et al., 2017; Wang et al., 2018). Indeed, lncRNAs not only affect cell apoptosis but also change cell fibrosis in DN. Although MALAT1 has been reported to regulate renal tubular epithelial pyroptosis in DN progression (Li et al., 2017a), the mechanism of MALAT1 in regulating DN still needs further study. Our result showed that MALAT1 could absorb miR-2355-3p to accelerate HG-induced renal fibrosis. miR-2355-3p regulates intervertebral disc degeneration by targeting ERFFI1 (Guo et al., 2019b), while it has not been largely reported in DN progression. Thus, our research expands the research of MALAT1 in DN progression and complements the research of miR-2355-3p in diseases.

IL6ST, also known as gpl30, is a signal subunit of the IL-6 receptor. When the IL-6 receptor binds to its ligand, it binds to another membrane glycoprotein gpl30 to enhance the cytokine response (Kidd et al., 1992). In target cells, only the IL-6 or IL-6 receptor (IL-6R) could not transmit the signal. Only when the IL-6/IL-6R complex binds to gp130 does signal transduction begin. The dimerization of gp130 results in the activation of tyrosine kinase Janus kinase-1 (JAK1), which binds to the cytoplasmic components of gp130. After phosphorylation, JAK1 phosphorylated 5 tyrosine residues in gp130 cells. This leads to the activation of a variety of intracellular signaling pathways, including MAP kinase and PI3 kinase pathways, as well as STAT1 and the STAT3 pathways (Schmidt-Arras and Rose-John, 2016). We predicted and verified that IL6ST was a target of miR-2355-3p. In addition, our result found that knockdown of IL6ST inhibited the phosphorylation of STAT3 and NF-kB protein levels in HG-treated HK-2 cells, while overexpression of IL6ST promoted the phosphorylation of STAT3 and NF-kB protein levels. Moreover, overexpression of miR-2355-3p downregulated IL-6ST mRNA and protein in HG-treated HK-2 cells. Some literature studies reported that the IL-6 response was mediated by a IL6ST-STAT3–dependent mechanism (Garbers et al., 2015; Wang et al., 2019). In STZ-induced type 1 diabetic mice and T2D db/db mouse kidney models, diabetes increased the expression of TGF-β1 and induced renal fibrosis through the Smad-independent pathway, the MAP kinases, or the Akt activation (Zhao et al., 2018; Zheng et al., 2019). It can trigger the transformation of the innate immune response to the adaptive immune response. In addition, it is involved in local tissue remodeling and immune cell infiltration (Feigerlovã and Battaglia-Hsu, 2017). The IL-6/STAT3 signaling pathway has been investigated in gastric cancer research, and activation of IL6ST enhances carcinogenesis (Chen et al., 2020). Although it has been previously reported that IL-6 stimulates STAT3 phosphorylation in acute kidney injury, the mechanism of IL-6 in the STAT3/NF-kB signaling pathway remains to be investigated (Ranganathan et al., 2013). As a pro-inflammatory cytokine, IL-6 may activate gp130 homologous dimer (IL6ST), thereby activating the JAK/STAT signaling pathway. Our results suggested that IL-6 affects renal fibrosis by acting on IL6ST in DN progression, and the IL6ST/STAT3/NF-kB signaling pathway plays an important role in DN progression. In conclusion, these findings demonstrated that lncRNA MALAT1 might enhance renal fibrosis in diabetic rats and cell damage in HG-induced HK-2 cells *via* the miR-2355-3p/IL6ST axis.

## Data Availability

The original contributions presented in the study are included in the article/Supplementary Material; further inquiries can be directed to the corresponding author.

## Supplementary Material

**SUPPLEMENTARY FIGURE 1** | MiR-2355-3p levels were downregulated in kidney and peritoneal tissues of STZ-induced diabetic rats. LNA-anti-miR-2355-3p and scramble (a negative control of LNA-anti-miR-2355-3p) were injected intraperitoneally into STZ-induced diabetic rats, and then RT-qPCR was used to detect miR-2355-3p levels in kidney, heart, liver, bladder and peritoneum tissues. ***P* < 0.01 versus scramble group.
