# Supplementary figures and images for: lncRNA MALAT1 Promotes Renal Fibrosis in Diabetic Nephropathy by Targeting the miR-2355-3p/IL6ST Axis
Source: Front Pharmacol. 2021 Apr 29;12:647650. doi: 10.3389/fphar.2021.647650 (PMC8117091; doi:10.3389/fphar.2021.647650)

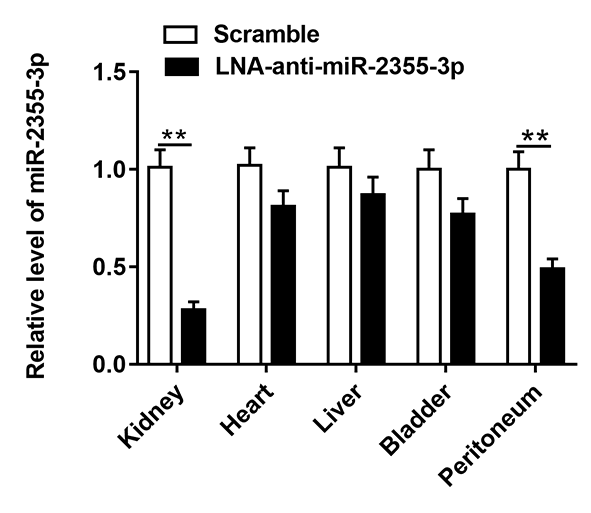

Supplement: Supplementary file 1 [file Image1.TIF]
